# Effects of Zinc Oxide Nanoparticles Synthesized Using *Aspergillus niger* on Carbapenem-Resistant *Klebsiella pneumonia In Vitro* and *In Vivo*

**DOI:** 10.3389/fcimb.2021.748739

**Published:** 2021-11-16

**Authors:** Elsayim Rasha, Manal M. Alkhulaifi, Monerah AlOthman, Ibrahim Khalid, Elnagar Doaa, Khatab Alaa, Manal A. Awad, Mohnad Abdalla

**Affiliations:** ^1^ Department of Botany and Microbiology, College of Science, King Saud University, Riyadh, Saudi Arabia; ^2^ Department of Zoology, College of Science, King Saud University, Riyadh, Saudi Arabia; ^3^ College of Science, King Saud University, Riyadh, Saudi Arabia; ^4^ Department of Medicine, Vascular Biology Center, Medical College of Georgia at Augusta University, Augusta, GA, United States

**Keywords:** zinc oxide nanoparticle, antimicrobial resistance, biological synthesis, carbapenem-resistant *Klebsiella pneumoniae* (KPC), *Aspergillus niger*, wound recovery

## Abstract

Currently, the mortality rate in Saudi Arabia’s ICUs is increasing due to the spread of *Klebsiella pneumoniae* carbapenemase (KPC)-producing bacteria. This study was carried out to evaluate the ability of biologically synthesized zinc oxide nanoparticles (ZnO-NPs) using *Aspergillus niger* to overcome carbapenem-resistant *K. pneumoniae* (KPC) *in vitro* and *in vivo*. ZnO-NPs were synthesized *via* a biological method and characterized using UV–Vis spectroscopy, Zetasizer and zeta potential analyses, x-ray diffraction spectroscopy, Fourier transform infrared spectroscopy, scanning electron microscopy (SEM), and energy-dispersive x-ray spectroscopy (EDX). *In vitro* sensitivity of KPC to ZnO-NPs was identified using the well diffusion method. The minimum inhibitory concentration (MIC) and minimum bactericidal concentration (MBC) were determined by a macro-dilution method. The morphological alteration of KPC cells after ZnO-NPs treatment was observed by SEM. The *in vivo* susceptibility of KPC cells to ZnO-NPs ointment was evaluated using wound healing in experimental rats. The chemical characterization findings showed the formation, stability, shape, and size of the synthesized nanoparticles. The MIC and MBC were 0.7 and 1.8 mg/ml, respectively. The *in vivo* results displayed reduced inflammation and wound re-epithelialization of KPC-infected rats. These findings demonstrated that ZnO-NPs have great potential to be developed as antibacterial agents.

## Introduction

*Klebsiella pneumoniae* is a Gram-negative, encapsulated, non-motile, rod-shaped opportunistic pathogen. It causes a wide range of hospital-acquired infections, such as wound infection, bacteremia, pneumonia, and urinary tract infections, particularly in immunocompromised people (Rani et al., 2017).

*K. pneumoniae* carbapenemase (KPC)-producing *Enterobacteriaceae* infection may be associated with treatment failure and increased mortality (Toledo et al., 2015). It is increasingly recognized as a serious public health concern worldwide. Thus, new strategies and ecofriendly drugs to eradicate KPC and to control multidrug-resistant bacterial strains need to be developed (Padalia et al., 2015). Among *Enterobacteriaceae*, carbapenemases are more prevalent in *K. pneumoniae* isolates, which usually cause hospital-acquired infections and outbreaks in Saudi Arabia. Alotaibi found that *K. pneumoniae* (63%) is more frequently isolated compared with *Escherichia coli* (55%) in a tertiary care hospital in Riyadh (Alotaibi, 2019).

International travel is the primary route of spread of KPC in the Gulf Cooperation Council (Saudi Arabia, United Arab Emirates, Oman, Kuwait, Qatar, and Bahrain). The strong economies of these countries have led to the arrival of large numbers of migrants to obtain work and medical care. In addition, millions of Muslims visit Saudi Arabia for the Hajj and other religious events every year, which further promotes the spread of KPC (Sonnevend et al., 2015).

In Saudi Arabia, the high incidence of KPC may be due to the large number of pilgrims, visitors, and migrant workers from endemic countries such as Turkey, India, and Pakistan every year (Liao et al., 2018). Surveys such as that conducted by Faiz and Khan have shown that the incidence of KPC among Makkah hospitals is about 48.4% from other carbapenem-producing organisms (Aftab Faiz, 2016).

Synthesis of nanoparticles is mediated by many methods such as physical, chemical, and biogenic methods (Afifi et al., 2015). The physical and chemical methods have many advantages involved in the attraction of nanoscale particles and formation of large, well-defined, and stable nanostructures. The physical method’s disadvantages include the use of costly equipment, high temperature and pressure, and large space area for setting up of machines. The chemical method’s disadvantages include the use of toxic chemicals that are hazardous for humans and the environment (Chandrasekaran et al., 2016).

Biosynthesis of nanoparticles refers to the synthesis of nanoparticles using plants or microorganisms. Nanoparticles from such “green synthesis” have been used in the field of drug, gene delivery, and various medical treatments including antimicrobial, anticancer, anti-inflammatory, antiaging, antioxidant, and anti-biofilm inhibition (Castro-Aceituno et al., 2016). Oxide nanoparticles synthesized using eukaryotic organisms such as fungi are beneficial because of their ability to produce a large amount of enzymes (Castro-Longoria et al., 2011).

Several attempts have been made to evaluate and study the antimicrobial activity of zinc oxide nanoparticles (ZnO-NPs). In October 2020, a group of researchers studied the antimicrobial activity of ZnO-NPs synthesized using aqueous extracts of pomegranate leaves and flowers designated as ZnO-NPs-PL and ZnO-NPs-PF, respectively. They found that ZnO-NPs were effective against all their selected pathogenic strains including *K. pneumoniae*. They reported that both ZnO-NPs can effectively be used as alternative antibacterial agents (Ifeanyichukwu et al., 2020). Another study (Farzana et al., 2017) showed that ZnO-NPs possess strong antimicrobial activity and can promote the antimicrobial activity of *K. pneumoniae* and *E. coli* (Farzana et al., 2017).

ZnO appears to have strong potential to kill microorganisms, whereas ZnO-NPs present strong antibacterial activities on broad-spectrum pathogenic bacteria such as *Bacillus subtilis*, *Staphylococcus aureus*, *E. coli*, *E. coli* O157:H7, *Salmonella enteritidis*, *S. typhimurium*, *Pseudomonas fluorescens*, and *Listeria monocytogenes*. ZnO-NPs exert their mode of action through inhibiting bacterial growth and increasing membrane permeability, thereby affecting the synthesis of hydrogen peroxide, cell wall penetration, and disorganization of bacterial membrane (Huh and Kwon, 2011; Ravushankar and Jamuna, 2011).

## Results

### Characterization of ZnO-NPs Synthesized Using *Aspergillus niger*

**Figure 1:**
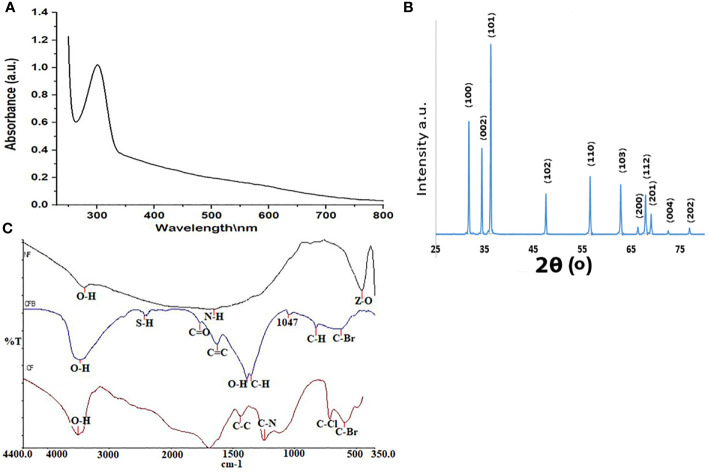
**(A)** UV–Vis spectra of ZnO-NPs. **(B)** XRD of ZnO-NPs. **(C)** FTIR of ZnO-NPs.

The results of UV–Vis absorbance spectrophotometry (wave range 200–800 nm) of the synthesized ZnO-NPs using *Aspergillus niger* showed an absorbance peak at 304 nm (**Figure 1A**). The x-ray diffraction (XRD) pattern of ZnO-NPs showed that the peaks of ZnO-NPs appeared at 2θ of 31.77°, 34.44°, 36.26°, 47.55°, 56.60°, 62.88°, 66.38°, 67.96°, 69.09°, 72.98°, 81.64°, 92.80°, 95.32°, and 98.67°, which corresponded to the (100), (002), (101), (110), (103), (112), (200), (201), (004), and (202) lattice planes (**Figure 1B**). To determine the functional groups responsible for the synthesis of ZnO-NPs and to compare the functional groups in *A. niger* that mediate the synthesized nanoparticles before and after calcination, we used Fourier transform infrared spectroscopy (FTIR) in the range of 400–4,000 cm^−1^. FTIR of ZnO-NPs according to **Figure 1C**, which presented the sharp peak at 3,482 and 3,401 cm^−1^, corresponds to the O-H strong group, which is found in ZnO-NPs before and after burning at 400°C (calcination) and the aqueous extract of *A. niger*. The ZO group and N-H medium, bend amine group were detected only in ZNO-NPs before calcination. After calcination showed S-H weak, stretching thiol, C=O stretch, α,β-unsaturated aldehyde ketone, C-H medium, rock alkanes, C-N medium, stretching aliphatic amines, C-H medium, wag alkyl halides and C-Br medium, and stretching alkyl halides. The previous functional groups in ZnO-NPs after calcination make them effective against the tested bacteria. The aqueous extract of *A. niger* has several functional groups that are responsible for the synthesis of ZnO-NPs groups, including C=O, C-C, C-N, C-Cl, and C-Br groups.

**Figure 2:**
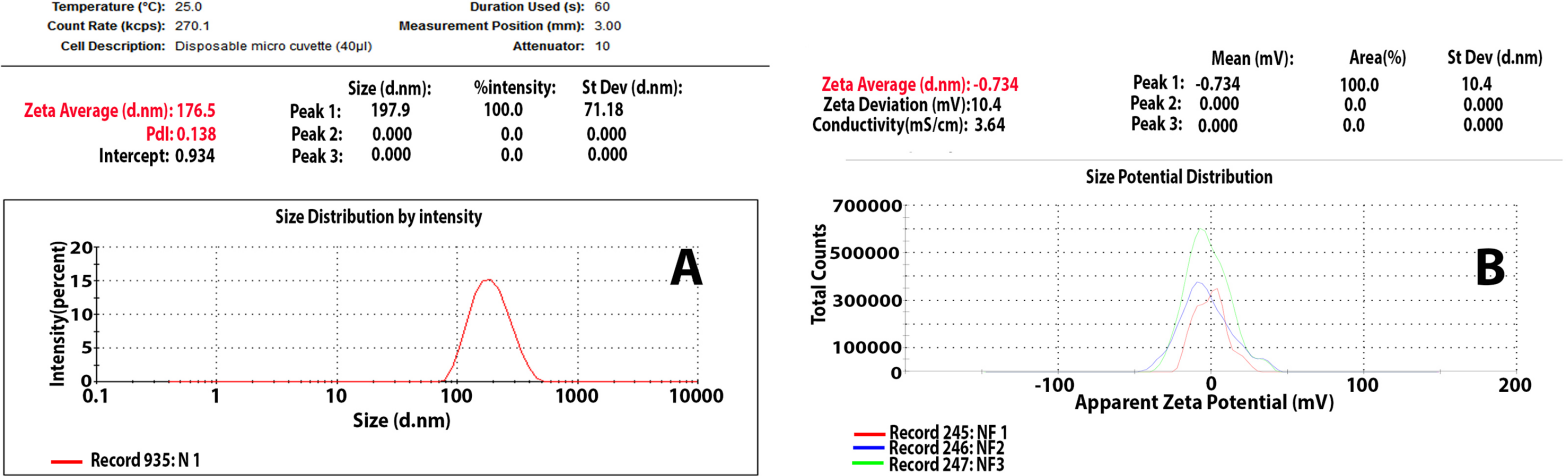
**(A)** Zeta size and **(B)** zeta potential of ZnO-NPs.

The measured zeta potential value of biosynthesized ZnO-NPs is shown in **Figures 2A, B**, and the mean Z-average diameter (nm) of the ZnO-NPs and the size distribution were observed to be 176.5 nm for zeta size and −0.734 mV for zeta potential. The size distribution profile was 100% and 0%.

**Figure 3:**
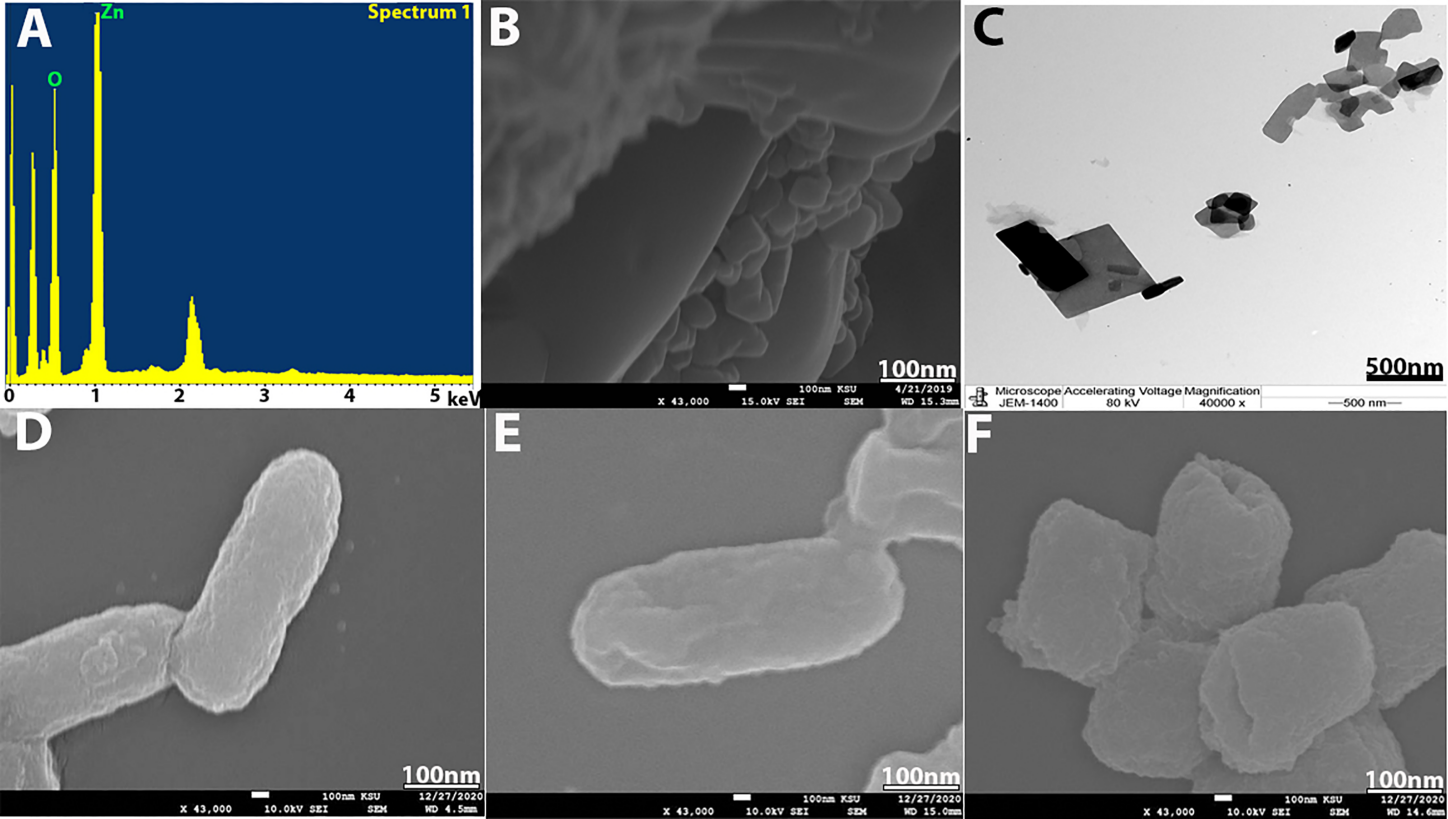
**(A)** EDX analysis. **(B)** SEM image of ZnO-NPs. **(C)** TEM image of ZnO-NPs. **(D)** SEM of bacterial control. **(E)** KPC treated with imipenem at 500 mg/ml and **(F)** KPC treated with ZnO-NPs.

SEM and energy-dispersive x-ray spectroscopy (EDX) were conducted to confirm the formation of nanoparticles and their elemental composition. SEM was used to analyze the structure of ZnO-NPs that were formed. The SEM image in **Figure 3B** shows that ZnO-NPs have an irregular, individual, quaternary shape, and most of them presented with aggregate shape with a smooth surface and were apparently devoid of cracks. The elemental analysis of ZnO-NPs for zinc and oxygen components showed 61.63% zinc and 38.37% oxygen (**Figure 3A**). TEM also appeared irregular, individual, and of quaternary shape, and the size of the synthesized nanoparticles by both TEM and SEM is about 82–176 nm (**Figure 3C**).

### Antibacterial Activity

**Figure 4:**
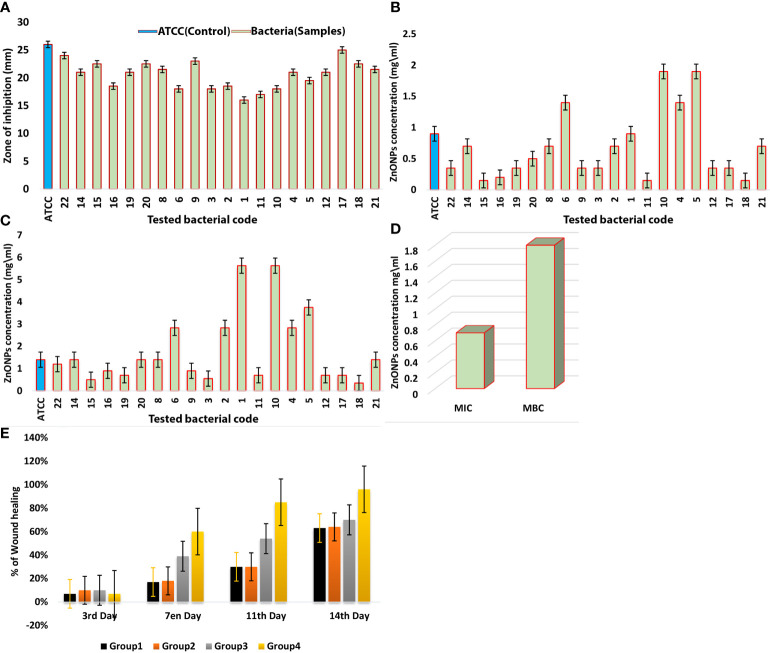
**(A)** ZOI (mm) of ZnO-NPs against *Klebsiella pneumoniae* (ATCC 700603) and KPC bacteria. **(B)** MIC of ZnO-NPs against KPC and *Klebsiella pneumoniae* (ATCC 700603). **(C)** MBC of ZnO-NPs against KPC and *Klebsiella pneumoniae* (ATCC 700603). **(D)** Comparison between MIC and MBC. The percentage of mean. **(E)** Wound recovery in the wound area within 14 days of wounding in G-1 (infected and untreated control), G-2 (uninfected and untreated control), G-3 (infected and treated with imipenem), and G-4 (infected and treated with ZnO-NPs).

The zone of inhibition (ZOI) was determined using an agar well diffusion method. The results showed the promising outcomes of the synthesized ZnO-NPs against tested bacteria (KPC). All tested KPC and *K. pneumoniae* (ATCC700603) as control exhibited high sensitivity to ZnO-NPs (20.8 ± 2.7 mm) at 7.5 mg/ml concentration (**Figure 4A**). The minimum inhibitory concentration (MIC) was determined by the macro-dilution method in culture broth, and the minimum bactericidal concentration (MBC) was obtained by the agar dilution method. **Figures 4B**–**D** and **Table 1** present the experimental data of MIC and MBC for all tested KPC. The mean score for MIC was 0.7 mg/ml, and that for MBC was 1.8 mg/ml.

**Table 1:** Comparison between MIC and MBC.

	MIC mg/ml	MBC mg/ml
Mean ± Std. Deviation	**0.7 ± 1.79**	**1.8 ± 1.56**

### SEM for Bacterial Cell Morphology

SEM was used to study the bacterial cell morphology changes. From **Figures 3D**–**F**, we can see that KPC cells treated with ZnO-NPs showed significant changes in the bacterial cell, including severe damage, decreased size, and change in shape from a rod shape to a slightly coccus shape. Moreover, multiple dents were observed on the cell surface. However, the untreated KPC cells and those treated with imipenem showed no morphological changes. The SEM findings are consistent with those of previous studies (Hameed et al., 2016; Liu et al., 2017; Sharma et al., 2019).

### ZnO-NPs Improve KPC-Infected Wound Healing in Rats

**Figure 5:**
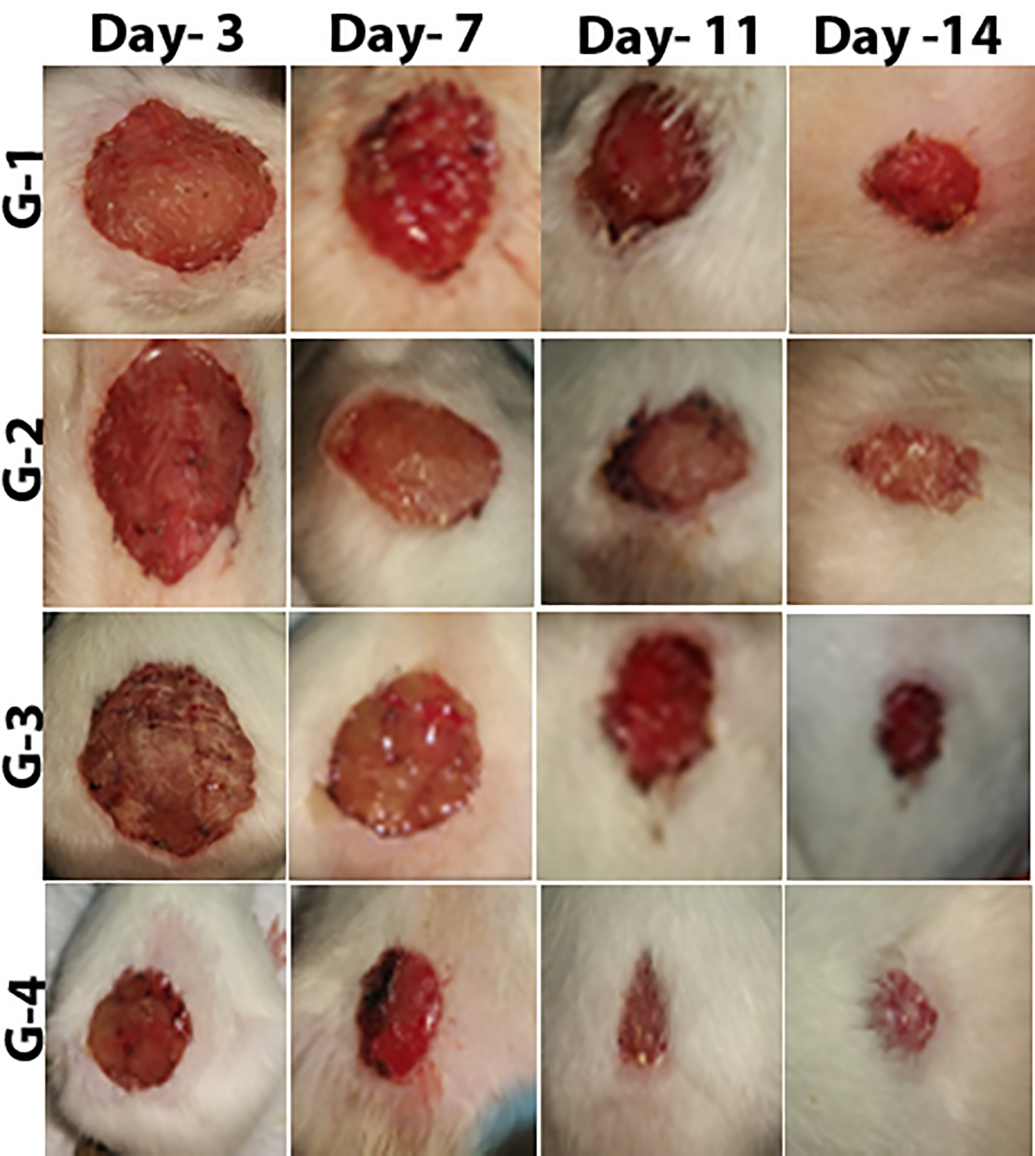
Stages of wound recovery in the wound area within 14 days of wounding in Group G-1 (infected and untreated control), G-2 (uninfected and untreated control), G-3 (infected and treated with imipenem), and G-4 (infected and treated with ZnO-NPs).

Wound healing evaluation was based on the change of fresh wounds of rats and the degree of closure on days 3, 7, 11, and 14 after wounding (**Figure 5**). Group-1 (G-1), the infected and untreated control group, showed severe tissue inflammation with purulence on the wound surface on all days. Group-2 (G-2), the uninfected and untreated control group, showed tissue inflammation on days 3, 7, and 11 with slight bleeding on day 7. Group-3 (G-3), the group infected and treated with imipenem ointment, showed severe inflammation on days 3 and 7, slight bleeding, and a thick mixture of layers of organisms and purulence on day 11. However, the tissue damage and inflammation were recovered on day 14. Group-4 (G-4), the group infected and treated with ZnO-NPs ointment, showed severe tissue inflammation with slight bleeding on days 3 and 7, which disappeared on day 11 with significant improvement of healing on day 14. Statistical analyses of the mean percentage of wound healing on day 14 after wounding showed that G-1 displayed comparable wound healing rate (63%) with G-2 (64%), G-3 exhibited moderate healing rate (70%), and G-4 presented promising results (96%) (**Table 2**; a histogram of **Table 2** is shown in **Figure 4E**).

**Table 2:** Percentage of mean wound recovery in the wound area within 14 days of wounding in G-1 (infected and untreated control), G-2 (uninfected and untreated control), G-3 (infected and treated with imipenem), and G-4 (infected and treated with ZnO-NPs).

Groups	Days			
	3rd Day in%	7th Day in%	11th Day in%	14th Day in%
Group 1	7 ± 2.739	17 ± 2.739	30 ± 5.000	63 ± 5.701
Group 2	10 ± 5.000	18 ± 6.708	30 ± 7.071	64 ± 9.618
Group 3	10 ± 3.536	39 ± 8.944	54 ± 4.183	70 ± 6.124
Group 4	7 ± 2.739	60 ± 5.000	85 ± 5000	96 ± 2.236

### General Histopathology of Wound Healing

**Figure 6:**
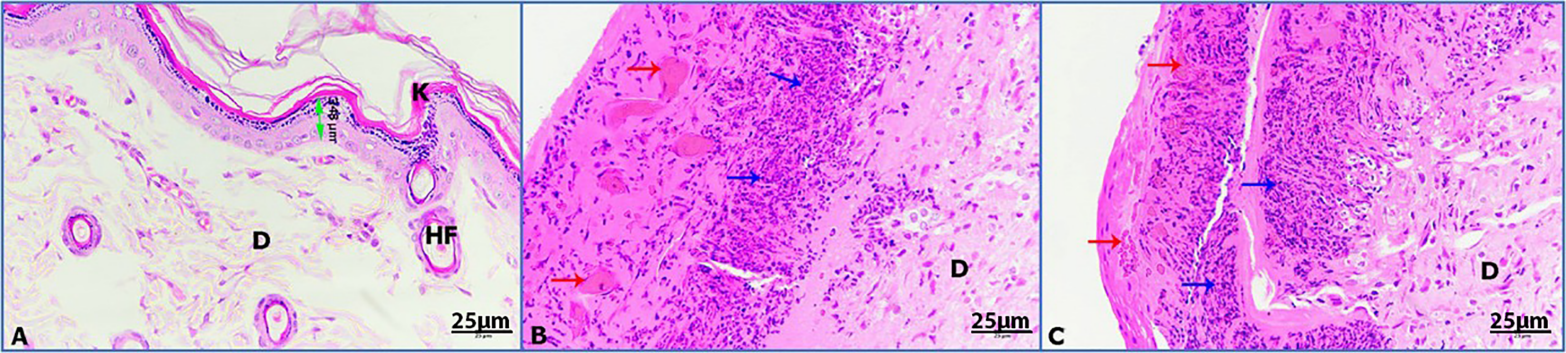
Photomicrographs of rat skin on day 3; (K) keratinized layer, (D) dermis, (HF) hair follicle, (double headed arrow) epidermis, (red arrows) hemorrhage, (blue arrows) granulomatous reaction. **(A)** Control skin, **(B)** untreated wounded skin, **(C)** untreated infected wounded skin (HE-400×).

Hematoxylin and eosin (H&E) was used to stain the stages of wound healing of the four groups of rats under investigation on days 3, 7, and 14. Unwounded control skin showed normal skin feature with thickened stratified squamous epithelia (348 µm) sheathed with keratinized layer and underneath with dermis layer of connective tissue (**Figure 6A**). Untreated wounded skin at 3 days post operation (POD) showed altered hemorrhage and granulomatous reaction of inflammatory cells (**Figure 6B**). Untreated infected wounded skin revealed more granulomatous reaction at 3 POD (**Figure 6C**).

**Figure 7:**
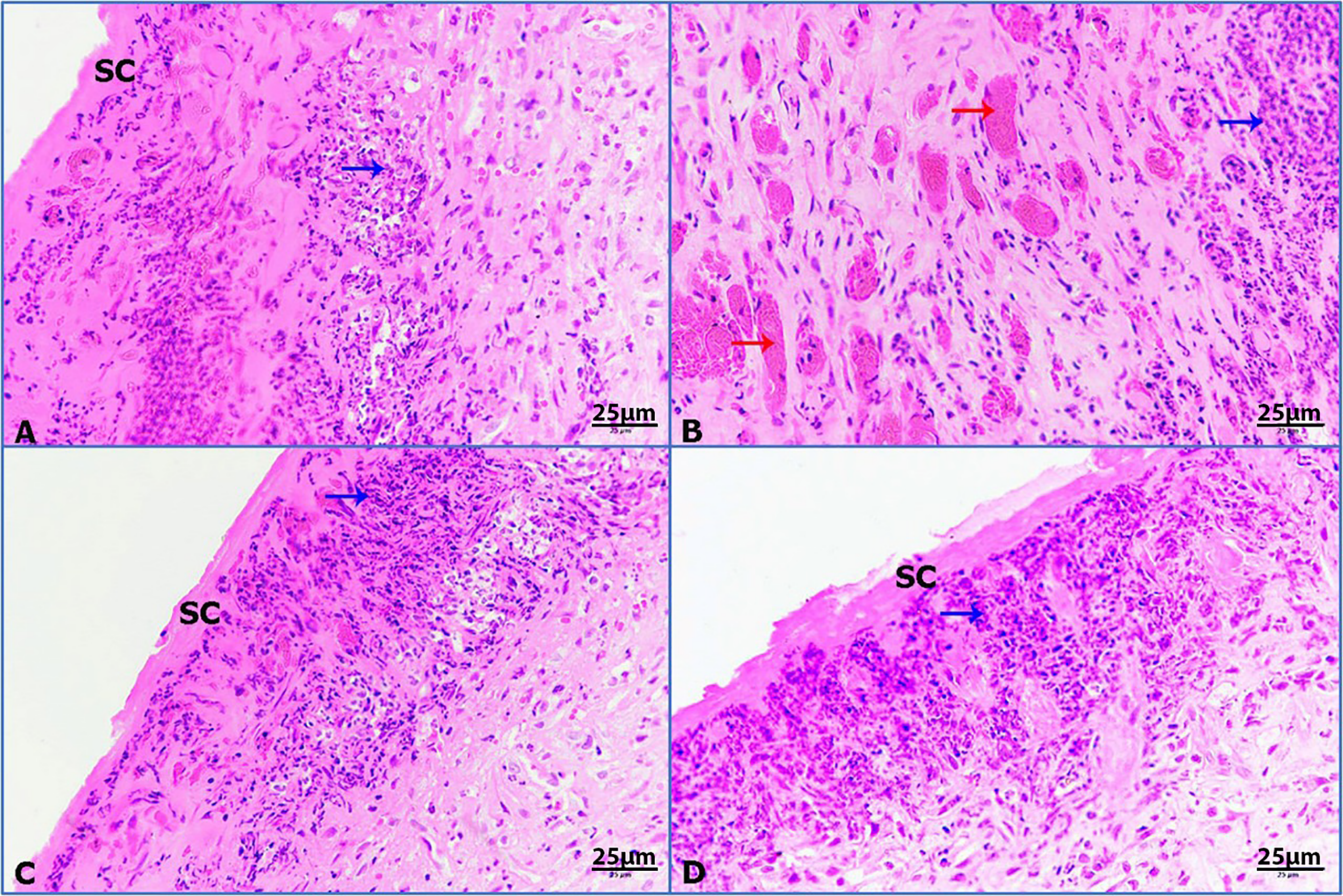
Photomicrographs of rat skin, (red arrows) hemorrhage, and (blue arrows) granulomatous reaction. **(A)** Untreated wounded skin, **(B)** infected untreated wounded skin, **(C)** infected wounded skin treated with imipenem, **(D)** infected wounded skin treated with ZnO-NPs (HE-400×).

Untreated wounded skin at 7 POD presented the primary formation of scab with inflammatory granulomatous reaction (**Figure 7A**). By contrast, infected untreated wounded skin showed increased incidence of hemorrhage foci and inflammation (**Figure 7B**). Moreover, infected wounded skin treated with imipenem exhibited the formation of scab and inflammation (**Figure 7C**). Furthermore, infected wounded skin treated with ZnO-NPs showed more scab formation and granulomatous reaction (**Figure 7D**).

**Figure 8:**
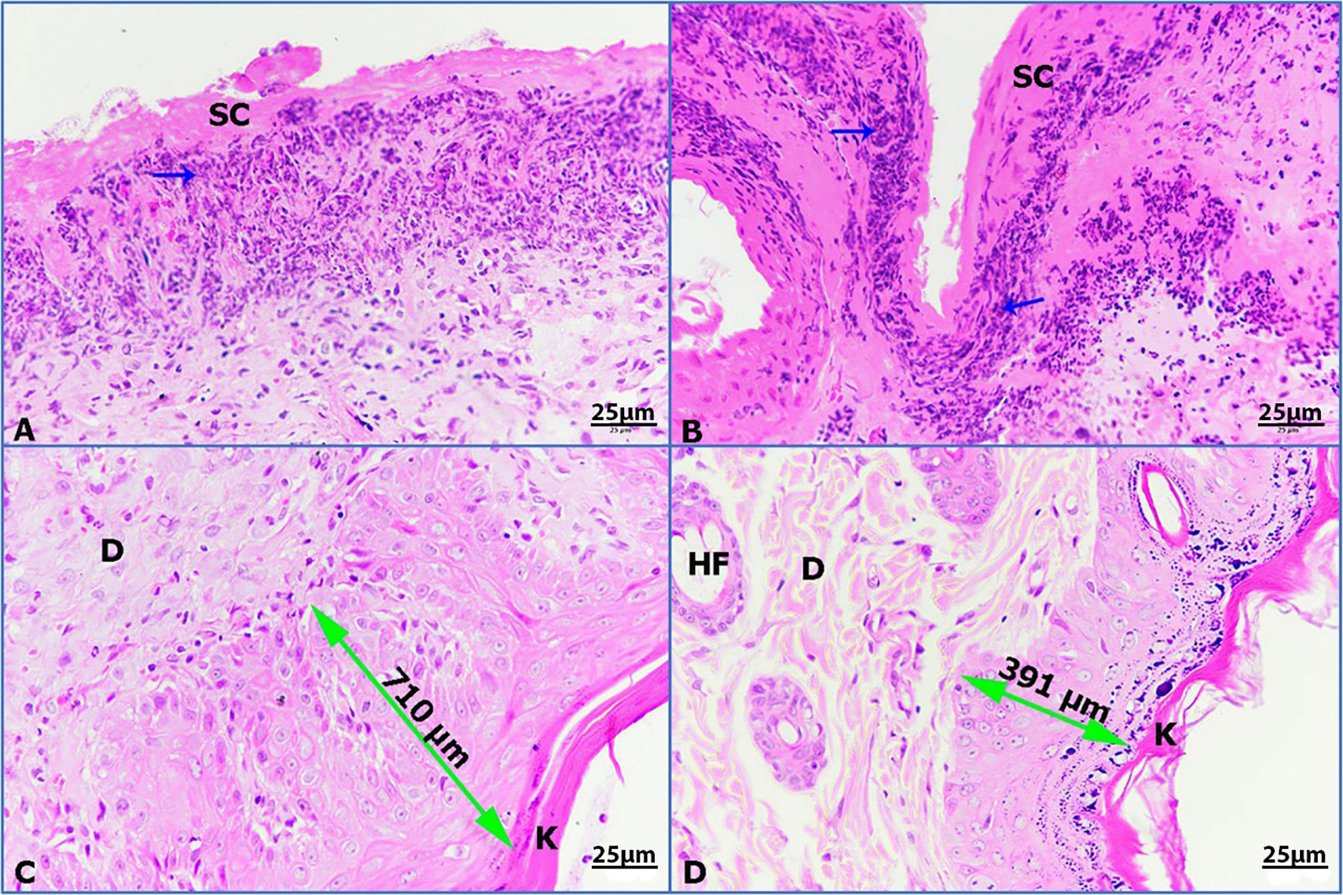
Photomicrographs of rat skin, (K) keratinized layer, (D) dermis, (HF) hair follicle, (double head arrow) regenerated epidermis, and (blue arrows) granulomatous reaction. **(A)** Untreated wounded skin, **(B)** infected untreated wounded skin, **(C)** infected wounded skin treated with imipenem, **(D)** infected wounded skin treated with ZnO-NPs (HE-400×).

Untreated wounded skin 14 days post wounding displayed a superficial scab and inflammatory granulomatous reaction (**Figure 8A**). However, infected untreated wounded skin showed more concentrated inflammation beneath the scab (**Figure 8B**). Additionally, infected wounded skin treated with imipenem showed thickened regenerated epidermis (710 µm) sheathed with keratinized layer (**Figure 8C**). Furthermore, infected wounded skin treated with ZnO-NPs showed marked improvement of wound with regenerated differentiated epidermis (391 µm) and more improved dermis (**Figure 8D**).

## Discussion

The initial objective of this study was to determine and develop a new antibacterial agent to manage bacterial infections due to KPC. The present study found that the ZnO-NPs synthesized from *A. niger* exhibited promising activity against KPC and the wound recovery. In reviewing the literature, no more *in vivo* studies of synthesized ZnO-NPs were found. The optical properties of ZnO-NPs were characterized using UV–Vis spectrophotometry. Zinc oxide formation was confirmed as the absorption peak (lambda max) was found near 304 nm. This result correlated with the result reported by Aldalbahi (Aldalbahi et al., 2020), who observed the maximum peaks of ZnO-NPs at 300 and 359 nm. The results of UV–Vis spectrophotometry confirmed the presence of ZnO-NPs. ZnO-NPs possess a negative zeta potential value of −0.734 mv, which indicated that these nanoparticles have high stability due to the electrostatic repulsive force (Chaudhuri and Malodia, 2017). For zeta size, ZnO-NPs were 176 nm in size. The XRD results are consistent with those of Hameed and others, Chaudhuri1 and Malodia, and Elsayim and others (Hameed et al., 2016; Chaudhuri and Malodia, 2017; Rasha et al. 2021). However, they differed from those in the study of Sharma and others (Aldalbahi et al., 2020), which showed several peaks at 2θ between 31.77° and 98.67° In terms of nanoparticle structure, ZnO-NPs showed a crystalline structure different from the structure of synthesized nanoparticles reported by Hameed and others and Suvarna and others (Yousef and Danial, 2012; Liu et al., 2017). XRD indicated that the synthesized nanoparticles are in their purest form and have a crystalline structure. A possible explanation for these results is due to their tight and strong diffraction peaks. FTIR was used to identify the various functional groups in the synthesized nanoparticles and the aqueous extract of *A. niger* as control. The functional group O-H was present in the aqueous extract of *A. niger* and ZnO-NPs before and after calcination. This result indicates that the O-H group is the reducing agent responsible for ZnO-NPs formation. Most of the functional groups detected, such as C-H, C=O, C-C, N-H, N-O, C-N, OH, and ZnO (**Figure 1C**), were similar to those obtained in previous studies (Senthilkumar and Sivakumar, 2014; Kavitha et al., 2017; Gautam, 2019). The size and morphology of ZnO-NPs were identified using SEM and TEM. The average size of ZnO-NPs was approximately 84-176 nm, and they had a quaternary shape. The results are consistent with the SEM findings of previous studies (Siddiquah et al., 2018a; Safawo et al., 2018; Alamdari et al., 2020). Interestingly, ZnO-NPs were found to have promising antibacterial activity. The result of EDX showed a high percentage of zinc more than the other elements; this indicates the purity and good quality of the synthesized ZnO-NP-s by *A. niger*. The mean ZOI was 20.8 mm for the tested KPC and 22.9 mm for ATCC (Cockerill et al., 2012). This finding corroborates the results of many researchers who obtained 16–27 mm of ZOI (Yousef and Danial, 2012; Jesline et al., 2015; Farzana et al., 2017; Sharma et al., 2017).

According to van Vuuren and Viljoen (2008), natural products with MIC values below 1.0 mg/ml are considered noteworthy. The mean MIC and MBC of ZnO-NPs were 0.7 and 1.8 mg/ml, respectively. This result is similar to that found by Yousef and Danial (2012). Their MIC result was 500 mg/ml against *B. subtilis*, *Bacillus megaterium*, *Sarcina lutea*, *K. pneumoniae*, and *Proteus vulgaris*. This result confirms that the biosynthesis of nanoparticles is better than other synthesis methods. According to the MIC and MBC results, ZnO-NPs achieved high stability and potency as antibacterial compounds. SEM analysis showed that ZnO-NPs that interact with KPC surface may lead to transformations in cell size and shape. These changes result in the disruption of the cell membrane and cell death (Kavitha et al., 2017), while no cell surface change appeared in the SEM image of both controls (positive and negative). Surprisingly, *in vivo* experiments showed that G-4, which was treated with ZnO-NP ointment, exhibited promising results in terms of fresh wound healing area, while G-3 showed moderate healing. These results may be due to the resistance of the tested bacteria to imipenem. G-1 and G-2 presented the lowest wound healing rate, which may be due to their dependence to immune response for recovery.

## Methodology

### Synthesis of ZnO-NPs by Using *Aspergillus* Species

**Figure 9:**
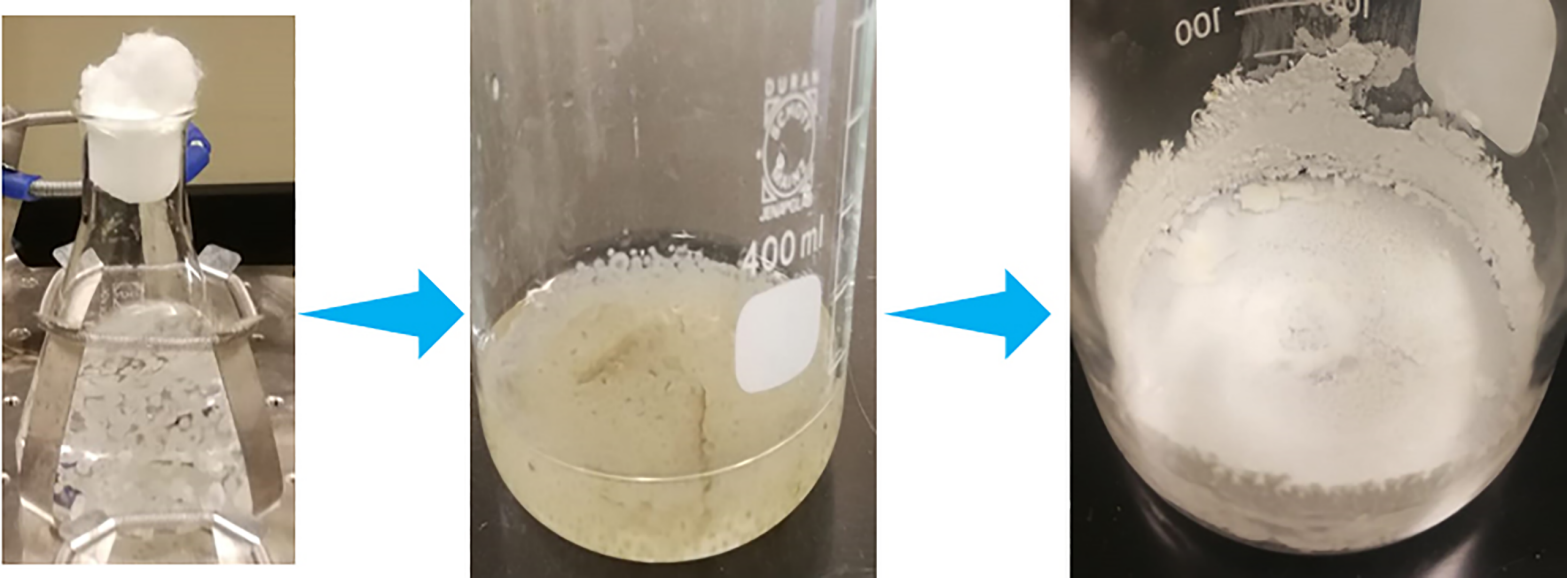
Synthesis steps of ZnO-NPs by using *Aspergillus niger*.

The biosynthesis of nanoparticles by *A. niger* was modified from two references to develop stable synthesized nanoparticles. *A. niger* (ATCC 16404 NA) was obtained from the Mycology Department of King Khaled Hospital and inoculated aerobically in Czapek Dox agar plate for 96 h at room temperature in an orbital shaker at 160 rpm. The fungal biomass was harvested by Whatman no. 1 filter paper to collect free fungal filtrate. Then, the filtrate was washed by centrifuging at 10,000 rpm for 20 min. Afterward, the sample was transferred to a 250-ml sterile flask, mixed with 200 ml of deionized water, and incubated at room temperature in a shaker incubator for 72 h at 200 rpm. Then, the sample was filtered using a Whatman no. 1 filter paper. The filtrate of *A. niger* was collected. To prepare ZnO-NPs, we prepared 100 ml of 0.25 mM solution of zinc nitrate and added 10 ml of *A. niger* filtrate. The sample was incubated in a shaker incubator at 180 rpm for 1 week at 37°C. Finally, white to yellow paste of ZnO-NPs was formed as shown in **Figure 9**. Then, it was stored until used (Rajan et al., 2016; Kalpana et al., 2018).

### Characterization of Synthesized ZnO-NPs

The formation of ZnO-NPs was confirmed by UV–Vis spectroscopy (UV-1800; Shimadzu UV Spectrophotometer, Kyoto, Japan), with a wavelength range of 200–800 nm at a resolution of 10 nm. The zeta size and potential measurement was performed using dynamic light scattering and Malvern Zetasizer nanoseries compact scattering spectrometer (Malvern Instruments Ltd., Malvern, UK), respectively. The histogram was developed by Zetasizer software (version 7.11) (Malvern Panalytical, Malvern, UK). The zeta potential was measured by using the folded capillary cell of Malvern. FTIR spectra were obtained in the range of 400–4000 cm^−1^ using a Nicolet FTIR instrument (Parkin Elmer, Spectrum BX, Waltham, UK) to identify and determine the different functional groups present in the *Aspergillus niger* filtrate before and after mixing with zinc nitrate, and the synthesized ZnO-NPs. The structure, crystalline nature, and composition of synthesized ZnO-NPs were analyzed by XRD. The formation, symmetry, size, and shape of nanoparticles were analyzed in powdered form by an x-ray diffractometer (Brucker-Discover D8, CUK-alpha, Sangamon Ave, Gibson, USA) of 2ɵ/min scan speed in the range 10°–100°. The size and composition of the synthesized nanoparticles were measured using SEM (JEOL model, JSM-761OF, Tokyo, Japan) operated at an accelerating voltage of 10 kV and equipped with an EDX detector. A small amount of dry ZnO-NPs was prepared as thin film on a carbon-coated copper grid. Then, the film was dried by placing it on the SEM grid under a mercury lamp for 5 min.

### *In Vitro* Microbial Susceptibility Testing of the Synthesized ZnO-NPs

Microbial susceptibility testing was performed by agar well diffusion technique. The tested bacteria (carbapenemase-producing *K. pneumoniae* [KPC]) were isolated as a retrospective sample from wounds of ICU patients at Prince Mohammed Bin Abdul Aziz Hospital-Al Madinah. Then, all samples were identified in the microbiology department of the hospital by using VITEK 2 system version 08.01. K*. pneumoniae* (ATCC 700603) was tested as control and was obtained from the College of Applied Medical Science, King Saud University. All bacterial samples were kept in nutrient agar slant and stored at 4°C until used (Omprakash and Sharada, 2015). The well plate agar diffusion method was carried out by inoculating the tested bacteria on nutrient broth overnight and adjusting to 0.5 McFarland turbidity standards. Then, each bacteria was streaked on a Mueller–Hinton agar (MHA) plate by swab. A sterile cork borer was used to form wells (6 mm in diameter) on the agar plates. Then, we added 7.5 mg of ZnO-NPs dissolved in 1 ml of deionized water to obtain a concentration of 7.5 mg/ml. Next, we added 0.5 ml of ZnO-NPs to each well in the inoculated culture plates and incubated them at 37°C overnight (Safawo et al., 2018). The microbial susceptibility was determined by measuring ZOI twice. The MIC was determined by the broth macro-dilution method according to the CLSI (Cockerill et al., 2012). The MBC was determined by obtaining loop full from MIC tubes, which did not show any visible growth and were inoculated on sterile MHA. The MBC result was recorded as the concentration where no visible growth was observed (Senthilkumar and Sivakumar, 2014).

### SEM for Bacterial Samples

Untreated KPC cells (negative control), those treated with imipenem (positive control), and those treated with ZnO-NPs (tested sample) were characterized by SEM to study the morphological cell alterations. The concentration of imipenem for positive control was 500 mg/ml, while for the tested bacteria, the MIC concentration was 0.5 mg/ml. The treated KPC cells were fixed with 2.5% glutaraldehyde in a phosphate buffer with pH 7.2. The samples were post-fixed in 1% osmium tetroxide, followed by dehydration through an ascending ethanol series to critical point drying and coating with Au–Pd (80:20) using a Polaron E5000 sputter coater (Quorum Technologies, Laughton, UK). The samples were analyzed by SEM (FEI Quanta 250) at an accelerating voltage of 25 kV using an SE detector (Kim et al., 2014; Kelly et al., 2017).

### *In Vivo* KPC Susceptibility for ZnO-NPs

All procedures including animals were approved by the Animal Care and Use Committee of King Saud University (Ethics Reference Number: KSU-SE-1978). Male Sprague–Dawley rats aged 12–14 weeks old were obtained from the Animal House of the College of Pharmacy, King Saud University, Riyadh, Saudi Arabia. Rats were anesthetized in accordance with the guidelines published by the University of California, San Francisco, Office of Research Institutional Animal Care and Use. Two-centimeter-thick cutaneous incisions were made on the dorsal area of rats under anesthesia (100 mg ketamine/kg of body weight and 5 mg xylazine/kg of body weight) (Kelly et al., 2017; Siddiquah et al., 2018b). The experimental rats were divided into four groups with each group having five rats: G-1 [wounded, infected with KPC, and untreated (positive control)], G-2 [wounded, uninfected with KPC, and untreated (negative control)], G-3 (wounded, infected with KPC, and treated with imipenem), and G-4 (wounded, infected with KPC, and treated with ZnO-NPs ointment) (Kelly et al., 2017). The treatment of wounded rats started 3 days after operation. The wound area was measured on days 3, 7, 11, and 14 post operation (Sivaranjani and Philominathan, 2016; Siddiquah et al., 2018b). The macroscopic images of the wound site were taken on days 3, 7, 11, and 14 using a Huawei Mate-9 camera. On day 14, the rats were sacrificed, and the skin tissues of the wound site were collected. H&E staining was performed for histological analysis (Zowawi et al., 2014). Tissue sections were checked under a light microscope (Nikon, Eclipse i80), and images were taken at different magnifications using a mounted Nikon digital camera (OXM 1200C; Nikon, Japan).

### Preparation of Gel-Based Ointments

Equal volumes of polyethylene glycol 400–2000 were added to ZnO-NPs and imipenem separately. Then, they were boiled at 65°C for 5 min. Ointment formulations (5 mg/ml) were prepared (Tiwari et al., 2018).

### Statistical Analysis

Statistical analysis was performed by using ANOVA with SPSS statistical software version 22 (SPSS Inc., Chicago, IL, USA). All results are presented as mean ± SD.

## Conclusion

This study was conducted to design new ways to treat carbapenem-resistant *Klebsiella pneumonia* and evaluate its activity *in vitro* and *in vivo*. In this study, we found promising results *in vivo* and *in vitro*, and we observed a correspondence between *in vitro* and *in vivo* results. The chemical characterization results of this study indicate that the biological synthesis of nanoparticles provided them with small size, suitable shape, long-term stability, and antibacterial activity. UV–Vis spectrophotometry at 304 nm confirmed the formation of ZnO-NPs. XRD and zeta potential results presented the purity and the crystalline form of ZnO-NPs. The O-H functional group played a significant role in the formation of ZnO-NPs, according to the FTIR findings. Zetasizer analysis found that the size of ZnO-NPs was 176 nm, and EDX results showed that the synthesized ZnO-NPs contained 61.63% zinc and 38.37% oxygen. The MIC of ZnO-NPs was 0.7 mg/ml, and the MBC was 1.8 mg/ml with 20.8 mm of ZOI. ZnO-NPs ointment presented interesting results in wound healing. These results support the aim of this study of using ZnO-NPs as antibacterial agent.

## Data Availability Statement

The original contributions presented in the study are included in the article/supplementary material. Further inquiries can be directed to the corresponding authors.

## Ethics Statement

The animal study was reviewed and approved by the Animal Care and Use Committee at King Saud University (Ethics Reference Number: KSU-SE-1978).

## Author Contributions

Conceptualization: MMA and ER. Data curation: ER. Funding acquisition: MAl. Investigation: MAl. Methodology: ER, ED, IK, MAA, and KA. Supervision: MAl. Writing—original draft: ER, MAb, and ED. Writing—review and editing, MAb. All authors contributed to the article and approved the submitted version.

## Conflict of Interest

The authors declare that the research was conducted in the absence of any commercial or financial relationships that could be construed as a potential conflict of interest.

## Publisher’s Note

All claims expressed in this article are solely those of the authors and do not necessarily represent those of their affiliated organizations, or those of the publisher, the editors and the reviewers. Any product that may be evaluated in this article, or claim that may be made by its manufacturer, is not guaranteed or endorsed by the publisher.
