# Corrigendum: Effects of Zinc Oxide Nanoparticles Synthesized Using *Aspergillus niger* on Carbapenem-Resistant *Klebsiella pneumonia In Vitro* and *In Vivo*

**DOI:** 10.3389/fcimb.2022.843577

**Published:** 2022-02-10

**Authors:** Elsayim Rasha, Manal M. Alkhulaifi, Monerah AlOthman, Ibrahim Khalid, Elnagar Doaa, Khatab Alaa, Manal A. Awad, Mohnad Abdalla

**Affiliations:** ^1^ Department of Botany and Microbiology, College of Science, King Saud University, Riyadh, Saudi Arabia; ^2^ Department of Zoology, College of Science, King Saud University, Riyadh, Saudi Arabia; ^3^ College of Science, King Saud University, Riyadh, Saudi Arabia; ^4^ Department of Medicine, Vascular Biology Center, Medical College of Georgia at Augusta University, Augusta, GA, United States

**Keywords:** zinc oxide nanoparticle, antimicrobial resistance, biological synthesis, carbapenem-resistant *Klebsiella pneumoniae* (KPC), *Aspergillus niger*, wound recovery

## Incorrect Affiliation

In the published article, there was an error in affiliation **3.** Instead of “King Abdullah Institute of Nanotechnology, King Saud University, Riyadh, Saudi Arabia”, it should be “College of Science, King Saud University, Riyadh, Saudi Arabia”.

The authors apologize for this error and state that this does not change the scientific conclusions of the article in any way. The original article has been updated.

## Publisher’s Note

All claims expressed in this article are solely those of the authors and do not necessarily represent those of their affiliated organizations, or those of the publisher, the editors and the reviewers. Any product that may be evaluated in this article, or claim that may be made by its manufacturer, is not guaranteed or endorsed by the publisher.

